# Effect of Acupuncture on Diabetic Neuropathy: A Narrative Review

**DOI:** 10.3390/ijms22168575

**Published:** 2021-08-09

**Authors:** Eunwoo Cho, Woojin Kim

**Affiliations:** Department of Physiology, College of Korean Medicine, Kyung Hee University, Seoul 02453, Korea; wg_eu4337@naver.com

**Keywords:** acupuncture, electro-acupuncture, diabetic neuropathy, pain

## Abstract

Diabetic neuropathy, a major complication of diabetes mellitus, refers to a collection of clinically diverse disorders affecting the nervous system that may present with pain. Although the number of patients suffering from severe neuropathy is increasing, no optimal treatment method has been developed yet. Acupuncture is well known for its ability to reduce various kinds of pain, and a number of studies have also reported its effect on diabetes mellitus; however, its effect and underlying mechanism against diabetic neuropathy are not yet clearly understood. In this review, ten and five studies performed in humans and animals, respectively, were analyzed. All studies reported that acupuncture significantly relieved diabetic neuropathy. ST36, BL13, BL20, SP6, and SP9 were the most widely used acupoints. Five studies used electro-acupuncture, whereas other studies used manual acupuncture. Furthermore, the effect of acupuncture was shown to be mediated through the various molecules present in the peripheral nerves and spinal cord, such as P65, GPR78, and TRPV1. Five studies reported side effects, such as swelling, numbness, and nausea, but none were reported to be serious. Based on these results, we suggest that acupuncture should be considered as a treatment option for diabetic neuropathy.

## 1. Introduction

According to a report by the International Diabetes Federation, 463 million adults were living with diabetes in 2019, and this number is expected to rise to 578 million by 2030 [1]. Diabetes causes many serious complications, such as ischemic heart disease, stroke, and kidney disease [2,3]. Among the many complications of diabetes, diabetic neuropathy, caused by damage to the peripheral and autonomic nervous systems, is by far the most prevalent, occurring in up to half of all individuals with diabetes [4]. Diabetic neuropathy refers to a collection of clinically diverse disorders that affect the nervous system due to hyperglycemia and microangiopathy [5,6]. The International Diabetes Federation estimated that one-third of the population will have diabetes in 2050, and half of those will have neuropathy without successful intervention [7,8]. The major patterns of nerve injury in diabetic neuropathy include distal symmetric polyneuropathy, small-fiber-predominant neuropathy, radiculoplexopathy, and mononeuropathy [9]. These syndromes cause substantial morbidity, increased mortality, and pain in affected patients [10]. In particular, neuropathic pain causes spontaneous pain, allodynia, hyperalgesia, hyperpathia, decreased physical activity, increased fatigue, and sleep problems, which negatively affect the quality of life [11,12].

Current approaches to the management of diabetic neuropathy focus on glycemic control, lifestyle modifications (such as diet and exercise), and drug-based pain management [13]. However, large meta-analyses have reported that glycemic control has minimal effect on neuropathy patients, especially in patients with type 2 diabetes [5], suggesting that focusing only on glycemic control may not be sufficient to attenuate diabetic neuropathy.

Various treatments, such as aldose reductase inhibitors, α-lipoic acid, and benfotiamine have been proposed as treatments for neuropathy. However, their efficacy is still under debate [14,15]. Furthermore, calcium channel α2δ ligands, serotonin and noradrenaline reuptake inhibitors (SNRI), and tricyclic antidepressants (TCAs) have been used, but comparative effectiveness studies to determine the best choice of medications are lacking [16,17,18,19,20].

Acupuncture has been widely used to treat various diseases. Its anti-inflammatory effect has been observed in several studies, including those on asthma [21,22], rheumatoid arthritis [23,24], epicondylitis [25,26], complex regional pain syndrome type 1, and vasculitis [27,28]. Furthermore, acupuncture is also known to treat different diseases of the nervous system as it helps reduce the doses of dopaminergic medications in patients with Parkinson’s disease [29] and relieve nerve dysfunction in the motor, non-motor, pre-motor, and autonomic nerves [30]. Moreover, acupuncture has been proven to be beneficial and effective for pain relief, such as for lower back pain [31], chronic tension-type headaches [32], and migraine prophylaxis [32]. Furthermore, various studies have reported that acupuncture can effectively modulate hyperglycemia in patients with diabetes mellitus [33,34]. Taken together, these results suggest that acupuncture could be considered as a therapeutic agent; however, to our knowledge, no review has summarized its effect and mechanisms on diabetic neuropathy.

In this review, by analyzing all clinical trials and animal studies that focused on the effect of acupuncture on diabetic neuropathy, we discuss its effect and its underlying mechanisms of action. Five animal studies and ten clinical trials are included in this review. Considering the increase in the number of patients with diabetic neuropathy worldwide, it is critical to analyze all studies published during the past decades for a better understanding of acupuncture and its underlying mechanism in treating diabetic neuropathy.

## 2. Results

### 2.1. Studies Conducted in Rodents

#### 2.1.1. Effect of Acupuncture in the Dorsal Root Ganglion (DRG) in Diabetic Neuropathy Alleviation

Among the five in vivo studies (Table 1), three focused on the DRG and two focused on the spinal cord. Shi et al. [35] observed the effect of electro-acupuncture (EA) on streptozotocin (STZ)-induced diabetic neuropathy in rats. Diabetes was induced by a single intraperitoneal injection of STZ, and rodents with a fasting blood glucose level of 16.65 mM and above were used in experiments. EA treatments were administered for 30 min, once a day, consecutively for 1 week. Alternate frequencies of 2 Hz and 100 Hz were used to stimulate acupuncture. For the experiments, the rats were divided into three groups. The first and second groups received EA treatment on ST36 and BL42, respectively. In the third group, needles were inserted into ST36 without any electrical stimulation. Their results showed that single EA treatment on bilateral ST36 significantly increased the paw withdrawal thresholds (PWT) at 2 h and 4 h compared to the control. Furthermore, multiple EA treatments also significantly increased PWT. This effect was initiated 30 min after EA treatment and lasted until day 5 compared to the control. However, the administration of EA on BL43 did not show any significant difference compared to the control. To clarify the mechanism of action of EA on diabetic neuropathy, they focused on nuclear factor kappa B (NF-κB) and cystathionine β synthase (CBS). They noticed that multiple EA treatments suppressed both p65 and CBS expression in the L4-6 DRG compared to the control. The sensitization of the DRG is considered a major factor in the development of aberrant pain. Moreover, in rodents experiencing neuropathic pain due to sciatic nerve injury, NF-κB significantly increased in the DRG [36], showing that NF-κB is involved in the development of neuropathy. Based on these results, it was suggested that EA could significantly attenuate diabetic neuropathy by suppressing NF-κB expression in the DRG.

Zhou et al. [37] also used the STZ-induced diabetic neuropathy model in rats to observe the effect of EA. EA was applied for 30 min on ST36 and BL60 with a frequency of 2 Hz for 1 week. The rats were divided into three groups: the first group received only vehicle, the second group received only STZ, and the third group received both STZ and EA. Their results showed that EA on ST36 and BL60 could significantly attenuate STZ-induced mechanical allodynia. PWT significantly decreased after 7 weeks of STZ administration and daily EA administration for 7 days. Although myelin disruption and dissolute axoplasm of sciatic nerve fiber and total protein of P2X purinoceptor 3 (P2X3) levels were not ameliorated by EA treatment, EA successfully suppressed the upregulated blood membrane protein levels of P2X3 receptor and p-protein kinase C (p-PKC) in the L4-6 DRG. P2X3 receptors, known to transduce nociceptive stimuli, are upregulated in the neuropathic pain state [38,39,40]. PKC is also an important modulator of pain and inflammation, and blocking PKC in rats with diabetic neuropathy has been related to the attenuation of mechanical hyperalgesia [41,42]. The intraperitoneal injection of P2X3 receptor agonists, αβ-methylene ATP [43], or PKC activators, phorbol 12-myristate 13-acetate [44] inhibited the anti-allodynia effect of EA and reversed the EA-induced downregulation of P2X3 receptors in the L4-6 DRG.

Pan et al. [45] also observed the effect of EA on STZ-induced diabetic neuropathy on rats with fasting blood glucose levels of 16.7 mM and above. EA was administered for 20 min, once a day, 6 days per week, for a total of 12 weeks. BL13, BL20, BL23, LI4, LR3, ST36, and SP6 acupoints were used. The frequency was set to 3 Hz. Only STZ injection without EA treatment was administered to the control group. EA increased the thermal sensitivity and motor conduction velocity (MCV) and sensory conduction velocity. Furthermore, in the STZ-only group, the nerve fibers were found to be damaged as the myelin sheaths were loose with irregular membranous masses, whereas EA treatment ameliorated these changes. In addition, EA decreased the proportion of apoptotic cells compared with the control. Most importantly, EA also lowered the levels of G-protein coupled receptor 78 (GPR78) and caspase-12, which are major factors in the endoplasmic reticulum stress (ERS) apoptosis pathway [46]. ERS apoptosis is regarded as the most important pathway under high-glucose conditions [47]. When cells are exposed to hyperglycemia, protein folding errors occur in the endoplasmic reticulum, leading to the accumulation of unfolded proteins and disruption of calcium homeostasis [48]. Under these conditions, ERS apoptosis is activated. Furthermore, in response to ERS, endoplasmic reticulum chaperones, such as the glucose-regulated protein 78, are upregulated to stabilize protein folding and prevent apoptosis [49]. Caspase-12 is the only member of the caspase family that participates in the ERS apoptosis pathway but not in the mitochondrial and death receptor apoptosis pathways [46,50]. This shows that EA could significantly inhibit the upregulation of GPR78 and caspase-12 in diabetic neuropathy.

#### 2.1.2. Effect of Acupuncture in the Spinal Cord in Diabetic Neuropathy Alleviation

Manni et al. [51] observed the effect of EA on thermal hyperalgesia induced by STZ injection. EA treatment of ST36 was applied for 30 min twice a week for 3 weeks. The frequency was set at 2 Hz. The STZ-injected group rats without any treatment were used as controls. Hyperalgesia to thermal pain was induced through the injection of STZ from the first to the fourth week. However, EA significantly increased the latency of the response to thermal pain compared to the control. Manni et al. focused on the hindpaw skin and spinal cord of rats to clarify EA’s mechanism of action [52,53]. Nerve growth factor (NGF) levels significantly increased after STZ treatment in both the skin and spinal cord of rats. However, compared to the control, EA treatment lowered the spinal cord’s NGF levels but not the skin’s. In addition, EA lowered the expression of tropomyosin receptor kinase A (TrkA) and pTyr496-TrkA, both NGF receptors, with the latter being the activated forms of TrkA in the skin and spinal cord. After STZ treatment, substance P (SP) levels were significantly increased in the skin and decreased in the spinal cord, but EA lowered that of the skin compared to the control. Transient receptor potential vanilloid 1 (TRPV1) was elevated in the skin and spinal cord after STZ administration. However, EA increased the TRPV1 levels in the skin and lowered the TRPV1 levels in the spinal cord. NGF is known to control spinal SP and TRPV1 [54,55,56], and it has been shown to be related to pain and diabetic neuropathy. Furthermore, EA restored the glutamic acid decarboxylase-67 protein, which synthesizes *gamma-*aminobutyric acid (GABA). GABA has been reported to be closely involved in the analgesic effect of EA [57] and in sensory dysfunctions induced by diabetes mellitus [58,59]. The results of this study showed that the effect of EA on thermal hyperalgesia is mediated by the spinal NGF signaling pathway through spinal neurotransmitters (e.g., GABA) and neuromodulators (e.g., SP and TRPV1).

After inducing diabetes through STZ, Tang et al. [60] studied Sprague–Dawley rats with fasting blood glucose levels of 11.1 mM and above. Acupuncture was administered for 20 min, once a day, for 2 weeks on BL13, BL20, and V23 acupoints. The mechanical withdrawal threshold and thermal withdrawal latency significantly increased after acupuncture treatment compared to the control. Moreover, acupuncture lowered the levels of inflammatory factors, G protein-coupled receptor 9, interleukin-1β, IL-6, and tumor necrosis factor-α compared to the control. In addition, the increased levels of glycosylated serum protein, triglyceride, total cholesterol, low-density lipoprotein, and decreased levels of high-density lipoprotein following STZ injection were regulated after acupuncture treatment. STZ increased spinal P2X purinoceptor 4 (P2X4) and microglia marker [61] expression, which was counteracted by acupuncture. Spinal cord microglia are important for the development and maintenance of diabetic neuropathic pain [62,63,64], and it has been reported that P2X4 receptors are expressed on microglia [65]. P2X4 receptors have been shown to be involved in mechanical allodynia induced by nerve damage. As such, blocking P2X4 receptors can reduce pain hypersensitivity [62,66].

**Table 1:** Summary of in vivo Studies.

Authors	Type of Animal	Diabetic Neuropathy	Acupuncture Treatment ① Acupuncture Types ② Acupoints ③ Retention Time ④ Treatment Duration (Sessions) ⑤ Frequency ⑥ Intensity ⑦ Control to Acupuncture		Findings
Manni et al., 2011 [51]	SD Rats Female	Single STZ, 65 mg/kg, i.p., >16.65 mM	①	EA	STZ decreased latency of response to heat stimuli one to four weeks after administration (*p* < 0.05 vs. naive). EA at ST36 significantly increased the heat latency time at 4 weeks (*p* < 0.05 vs. control). STZ increased NGF levels both in the skin and in the spinal cord. EA only lowered that of the spinal cord (*p* < 0.05 vs. control). STZ increased SP levels in the skin but decreased in the spinal cord. EA only lowered that of the skin (*p* < 0.05 vs. control). STZ increased TrkA and pTyr496-TrkA levels in the skin. EA significantly decreased both (*p* < 0.05 vs. control). STZ significantly increased TRPV1 levels in the skin and the spinal cord. EA increased the TRPV1 level in the skin (*p* < 0.05 vs. control), whereas it decreased in the spinal cord (*p* < 0.05 vs. control). STZ decreased GAD-67 protein content in the spinal cord and EA significantly restored it (*p* < 0.05 vs. control).
			②	ST36	
			③	30 min	
			④	3 weeks (6)	
			⑤	2 Hz	
			⑥	1.0–1.5 mA	
			⑦	STZ alone	
Shi et al., 2013 [35]	SD Rats Female	Single STZ, 65 mg/kg, i.p., >16.7 mM	①	EA	Single STZ injection-induced mechanical allodynia from week 2 to 6 (*p* < 0.01 vs. naive). Single EA treatment at ST36 significantly increased the mechanical allodynia from 2 h to 4 h compared to control (*p* < 0.05). EA on BL43 showed no significant effect compared to sham. Multiple EA treatment significantly increased the PWTs starting from 30 min to D5 (*p* < 0.05 vs. control). Multiple EA on BL43 did not show significant differences. Multiple EA treatment suppresses p65 and CBS expression in L4-6 DRG (*p* < 0.05 vs. control).
			②	ST36, BL43	
			③	30 min	
			④	One week (7)	
			⑤	2/100 Hz	
			⑥	1 mA	
			⑦	EA at ST36 without electrical stimulation	
Zhou et al., 2018 [37]	SD Rats Male	Single STZ, 35 mg/kg, i.p., ≥11.1 mM	①	EA	Single STZ injection induced mechanical allodynia 7 weeks after its injection (*p* < 0.01 vs. naive). EA treatment for 7 consecutive days on week 7 significantly attenuated mechanical allodynia (*p* < 0.01 vs. control). EA failed to improve myelin disruption and dissolute axoplasm of sciatic nerve induced by STZ. EA suppressed STZ induced upregulation of blood membrane protein levels of P2X3 receptor and p-PKC expressions in L4-6 DRG (*p* < 0.01 vs. control) Intraperitoneal injection of αβ-meATP or PMA inhibited the anti-allodynic effect of EA. PMA injection reversed downregulation of the plasma membrane protein levels of P2X3 receptors in the DRG.
			②	ST36, BL60	
			③	30 min	
			④	One week (7)	
			⑤	2 Hz	
			⑥	1–2 mA	
			⑦	STZ alone	
Pan et al., 2019 [45]	SD Rats Male	Single STZ, 50 mg/kg, i.p., ≥16.7 mM	①	EA	EA significantly increased the decreased heat sensitivity on week 14 (vs. control). EA significantly increased downregulated MCV and SCV (vs. control). MCV of naive group; 50.67 ± 10.71 m/s to 50.86 ± 11.04 m/s, control group; 45.00 ± 9.44 m/s to 20.63 ± 10.27 m/s, EA group; 43.84 ± 9.14 m/s to 30.26 ± 8.96 m/s. SCV of naive group; 51.26 ± 8.93 m/s to 48.32 ± 12.01 m/s; control group; 46.28 ± 11.65 m/s to 21.43 ± 11.51 m/s, EA group; 45.13 ± 9.49 m/s to 29.54 ± 9.39 m/s. EA ameliorated loose with irregular membranous masses myelin sheaths and damaged myelinated nerve fibers induced by STZ injection. EA decreased the upregulated proportion of apoptotic cells (vs. control). EA lowered mean level of GPR78 and Caspase-12. GRP78 of naive group; 0.21 ± 0.05, control; 0.48 ± 0.18, EA group; 0.29 ± 0.07. Caspase-12 of naive group; 0.22 ± 0.07, control; 0.48 ± 0.28, EA group; 0.26 ± 0.04.
			②	BL13, BL20, BL23, LI4, LR3, ST36, SP6	
			③	20 min	
			④	12 weeks (72)	
			⑤	3 Hz	
			⑥	-	
			⑦	STZ alone	
Tang et al., 2020 [60]	SD Rats Male	Single STZ, 35 mg/kg, i.p., ≥11.1 mM	①	Manual acupuncture	Acupuncture treatment increased STZ-induced lowered MWT and TWL at D14 (*p* < 0.001) and D7-D14 (*p* < 0.05 (D7) and *p* < 0.001 (D10, D14)), respectively. Acupuncture lowered the serum levels of CXCR3, IL-1β, IL-6 and TNF-α significantly increased after STZ injection (*p* < 0.001 vs. control) at D14. Acupuncture lowered the serum level of GSP, TG, TC, LDL-C, and elevated the level of HDL-C altered due to STZ injection (*p* < 0.001 vs. control) at D14. Acupuncture reduced the increased expression of spinal P2X4 and OX42 expression (*p* < 0.001 vs. control).
			②	BL13, BL20, V23	
			③	20 min	
			④	2 weeks (14)	
			⑤	-	
			⑥	-	
			⑦	STZ alone	

Abbreviations: αβ-meATP: αβ-methylene ATP; CBS: Cystathionine β Synthase; CXCR3: G protein-coupled receptor 9; DRG: Dorsal Root Ganglion; EA: Electro-Acupuncture; GAD-67: Glutamic Acid Decarboxylase-67; GPR78: G-Protein Coupled Receptor 78; GSP: Glycosylated Serum Protein; HDL: High-Density Lipoprotein; IL-1β: Interleukin-1β; IL-6: Interleukin-6; LDL: Low-Density Lipoprotein; MCV: Motor Conduction Velocity; MWT: Mechanical Withdrawal Threshold; NGF: Nerve Growth Factor; P2X3: P2X purinoceptor 3; P2X4: P2X purinoceptor 4; PMA: Phorbol 12-Myristate 13-Acetate; p-PKC: p-Protein Kinase C; PWT: Paw Withdrawal Threshold; SCV: Sensory Conduction Velocity; SD: Sprague Dawley; SP: Substance P; STZ: Streptozotocin; TC: Total Cholesterol; TG: Triglyceride; TNF-α: Tumor Necrosis Factor-α; TrkA: tropomyosin receptor kinase A; TRPV1: Transient Receptor Potential Vanilloid 1; TWL: Thermal Withdrawal Latency.

### 2.2. Effects of Acupuncture in Studies Conducted in Humans

To observe the effect of acupuncture on diabetic neuropathy, Abuaisha et al. [67] recruited 44 participants with diabetic neuropathy. Among them, 29 were already receiving conventional treatments, such as anticonvulsants and tricyclic drugs. Acupuncture was applied to LI3, SP6, SP9, and ST36. After six sessions of acupuncture treatment for 10 weeks, the primary and secondary symptoms were significantly mitigated in 34 of the 44 participants (77%) compared to baseline. They defined primary symptoms as the most troublesome symptoms and secondary symptoms as the minor symptoms. Moreover, seven patients (21%) reported complete symptom relief. The 34 patients who saw improvements following acupuncture were followed up for 18 to 52 weeks, and 67% were able to stop or reduce their medications. However, there were no significant changes in neuropathy disability score (NDS), vibration perception threshold (VPT), or HbA1c levels during the treatment. It has been reported that the NDS is a sensitive marker of neuropathy disability [68] while the VPT also allows for the assessment of neural dysfunctions [69]. In this study, only one participant felt uncomfortable and withdrew after two sessions.

Jiang et al. [70] divided 90 participants into three groups to assess the effect of acupuncture on diabetic neuropathy. The trial was initiated when the blood sugar had been kept stable for more than 3 months, and medication for diabetic peripheral neuritis had been withdrawn for more than 2 weeks. The wrist–ankle acupuncture group was bilaterally treated with acupuncture on the acupoints on the wrist and ankle, while the body acupuncture group was treated with acupuncture. The control group participants were given an intramuscular injection of vitamin B1 and vitamin B12 once daily. Each group received a total of 21 sessions over 25 days. In the wrist–ankle acupuncture group, 56.67% of patients expressed “markedly relieved,” which referred to the decreased rate of the total evaluation score for the clinical symptoms after the treatment for above 65%, while the indexes of blood sugar, blood lipid, blood rheology, and nerve conductive velocity were within the normal range. Furthermore, 36.67% expressed “improved,” referring to the decreasing rate of the total evaluation score for the clinical symptoms after the treatment reached 25%–64%, and the indexes of blood sugar, blood lipid, blood rheology, and nerve conductive velocity were within the normal range. In the body acupuncture group, 56.67% expressed “markedly relieved,” and 33.33% expressed “improved.” In the control group, 23.33% reported “markedly relieved,” and 40% expressed “improved.” The specific levels of blood lipids, such as cholesterol, high-density lipoprotein, and low-density lipoprotein, significantly improved after treatment in the wrist–ankle acupuncture group and body acupuncture group compared to baseline. However, there was no significant difference in the triglyceride levels between the groups. The motor nerve conduction velocity (MNCV) of the median nerve and common peroneal nerve in the wrist–ankle acupuncture group and body acupuncture group, respectively, improved compared to baseline. Based on these results, Jiang et al. reported that acupuncture may improve diabetic neuropathy and that acupuncture treatment on the wrist–ankle could be more effective than on the body.

Dividing 65 participants into two groups, Zhang et al. [71] also analyzed the effect of acupuncture on diabetic neuropathy. They mentioned that all participants were given conventional treatment to maintain the fasting blood glucose below 7.0 mM and the 2 h blood glucose below 11.1 mM. The acupuncture group received acupuncture at 12 acupoints with auxiliary acupoints chosen based on traditional Chinese medicine diagnosis. Acupuncture was administered once a day for 25 min and manipulated twice in a total of 70 sessions over 3 months. The control group was orally administered with inositol 2 g a day three times. Sixteen (50%) and seven (21%) patients in the acupuncture and control groups, respectively, reported “marked relieved,” which referred to the disappearance of the subjective symptoms, with no abnormalities found in the examination of the nervous system. Moreover, seven and twelve participants in the acupuncture and control groups, respectively, responded with “improved,” which refers to the condition that the subjective symptoms had been alleviated or the affected area had been reduced, with improvements examined through nervous system examination. Finally, four and twelve patients in the acupuncture and control groups, respectively, responded with “failed”, which refers to the condition that the subjective symptoms had not been improved or even aggravated. Thus, the total effective rates (markedly improved + improved) were 87.5% and 63% in the experimental and control groups, respectively. The rates between the two groups were significantly different.

In a study [72] assessing the effect of acupuncture on diabetic neuropathy by Tong et al., acupuncture was applied once daily for 15 days to patients, who were divided into two groups. The acupuncture group received treatments on LI4, ST40, LI11, ST36, and SP6 to a depth of 1.2–2.3 cm, along with de qi inducement. In contrast, the control group received acupuncture to a depth of only 0.3 cm without de qi inducement. There was no significant glycemic difference between the two groups. However, there was a significant improvement in the F-wave minimum latency and MNCV in the tibial nerve and F-wave conduction velocity (FCV) in the median nerve compared to baseline. It has been reported that in patients with neuropathy, the F-wave index in the upper limb is significantly lower than that in healthy controls [73]. Acupuncture significantly relieved the severity and extent of numbness, spontaneous pain, and alterations in temperature perception of the lower extremities and the severity and extent of rigidity in the upper extremities. The higher the severity score, the worse the symptoms, and a higher score indicates that the pain is transmitted from the fingers, toes to wrists, ankles, elbows, and knees [72].

In the study by Garrow et al. [74], 45 patients with diabetic neuropathy were treated with real or sham acupuncture. Ten sessions of acupuncture treatment for 10 weeks were applied to the LR3, KI3, SP6, SP10, and ST36 acupoints. For the control, sham acupuncture was also performed in the same acupoints, but the needles did not penetrate the skin. Both treatments remained for 30 min, and manipulation was performed 15 min later. In the acupuncture group, patients showed more improvement than the sham group in the assessment of neuropathic symptoms and signs (LANSS) score, visual analog scale (VAS), pain intensity, measure yourself medical outcome profile (MYMOP) score, sleep problem scale (SPS), and the physical component of the short form 36 (SF-36). However, the SF-36 bodily pain score was more effective in the control group than in the acupuncture group. In the acupuncture group, two patients experienced adverse events, with one having chest pain related to a chronic heart condition, and another experiencing exacerbated leg pain.

Jeon et al. [75] reported that acupuncture treatment can relieve diabetic neuropathic pain. Acupuncture was administered at Ex-LE10 (four points of needle insertion), LR3, GB41, GB39, ST36, GB34, and SP6 acupoints through 12 sessions of acupuncture treatments over 4 weeks. Treatments were maintained for 22–28 min, and de-qi was induced by manipulating the needle for 10 min. They checked the total symptom score (TSS) using the Michigan neuropathy screening instrument (MNSI). The TSS scores did not significantly change, but the MNSI scores improved from 6.33 ± 1.31 to 4.33 ± 3.00.

In a study by Bailey et al. [76], the participants were treated with acupuncture on Ex-LE10 (four points of needle insertion), ST32, ST37, ST42, SP7, SP9, KI1, KI3, KI9, LR4, LR7, GB34, and GB37 acupoints once per week for 10 weeks. The needles remained for 30 min, and de qi was induced in each session. The control group for acupuncture was not included in this study. Acupuncture significantly relieved the neuropathy total symptom scale (NTSS-6) scores for aching pain, burning pain, tingling, prickling, numbness, and allodynia. Although there was a mean difference in lancinating pain, this was not significant. The NDS value reflecting the established nerve damage did not change significantly after acupuncture treatment. The NDS assesses vibration threshold, temperature sensation, pinprick sensation, and the Achilles reflex [76]. In addition, the laser Doppler fluxmetry values were not significant, although there was a mean increase in the endothelial response to both acetylcholine and sodium nitroprusside. Laser Doppler fluxmetry measures the skin’s microvascular response to iontophoretically administered acetylcholine, which is nitric oxide-specific, [77] and sodium nitroprusside, which stimulates endothelial smooth muscle vasodilation [76].

Shin et al. [78] applied EA to treat diabetic neuropathy. A total of 106 of the 126 patients completed the treatment, and 98 patients were followed up for 8 weeks. Participants were treated with EA on ST36, GB39, SP9, SP6, LR3, GB41, and Ex-LE10 acupoints, depending on the site of pain. The EA frequency was set alternatively at 2 Hz and 120 Hz, and the participants were treated twice a week for 8 weeks. The control group did not receive any treatment. For the pain intensity numerical rating scale (PI-NRS) scores, patients in the EA group showed significant improvements compared to the control group at weeks 9, 13, and 17. In addition, the EA group showed significant improvement compared to the control group on the short-form McGill pain questionnaire, sleep interference scores, and the EuroQol-5 dimensions measured at week 9. The percentage of patients with improved scores in the patient global impression of change scale was greater in the EA group (82.5%) than in the control group (34.1%). However, there was no significant difference in nerve conduction and rescue medication consumption between the groups. A total of 9 and 19 participants in the acupuncture group and the control group withdrew from the treatment, and no significant difference was observed in the incidence of adverse events between the two groups.

To assess the effect of acupuncture on diabetic neuropathic pain, Chao et al. [79] divided the participants into three groups. The first group received acupuncture treatment once a week for 12 weeks, whereas the second group received treatment twice a week for the same amount of time. Only conventional care by physicians was available for the third group, which served as the control. The Jing well and Shu stream acupoints and other points were selected based on the diagnosis criteria in traditional Chinese medicine. In traditional Chinese medicine, there are five Shu points in each of the four limbs [80,81,82]. Jing (well), xing (spring), shu (stream), jing (river), and he (sea) are the sites where qi enters and exits the meridian, representing each organ [83]. The Jing (well) acupoints are located in the tips of the fingers or toes, while the Shu (stream) acupoints are located near the carpal or tarsal joints [80]. After 12 weeks of treatment, the follow-up period lasted for 6 weeks or more. Pain intensity was measured using an NRS, with pain levels being recorded every day via three questions using a 24 h recall period: average, worst, and least pain. Their results showed that acupuncture could significantly alleviate the average, worst, and least pain intensity compared to the control when measured on weeks 6 and 12. However, the quality of life, physical functioning, and neuropathic symptoms between the acupuncture and control groups were not significantly different. Fifteen cases of side effects, such as pain, swelling, numbness, cramps, and palpitation, in the left arm were reported, but all were mild.

Meyer-Hamme et al. [84] administered needle acupuncture, laser acupuncture, and placebo laser acupuncture to treat diabetic neuropathy. The Ex-LE10, Ex-LE12, and ST34 were selected as acupoints. In the laser acupuncture group, the laser was applied directly on the skin at 90° for 20 min on the same acupoints, with 685 nm wavelength, 35 mW optical power, 2.3 kJ/cm^2^ power density, and 500 μm spot diameter. In the placebo group, laser acupuncture was administered without the laser. All participants received 10 identical treatment sessions per week for 10 consecutive weeks. After the treatment, sural sensory nerve action potential (SNAP) was elevated in all three groups. However, the sensory nerve conduction velocity (SNCV) was elevated only in the manual and laser acupuncture groups. The mean tibial MNCV improved only in the manual acupuncture group, and no significant difference in motor nerve action potential (MNAP) was observed in all the groups. As for the mean changes in the diabetic neuropathy-related symptoms from baseline to after 15 weeks of treatment, the results obtained by patient-related outcome measures (assessed using 11-point NRS questionnaires) showed that acupuncture administration led to significant changes in all 12 items (neuropathic pain, hyperesthesia, cold sensation, heat sensation, burning pain, tingling, muscle cramps, numbness, unsteadiness of gait, impact on daily activities, impact on sleep quality, and frequency of symptoms). In the laser acupuncture group, 11 items (excluding hyperesthesia) showed significant differences. However, only nine items (excluding hyperesthesia, heat sensation, and muscle cramps) were altered in the placebo group. Minor hematomas by needle acupuncture were observed in approximately 22% (13 of 60 patients). Seven of the180 patients (4%) reported a temporary increase in neuropathic pain and decreased hypoesthesia, though the researchers reported that all adverse events were mild.

### 2.3. Outcomes in Clinical Studies

#### 2.3.1. Patients-Related Outcomes

In all ten clinical studies investigated (Table 2), the patient-related outcomes generally improved. Abuaisha et al. [67] reported an improvement in the primary and secondary symptoms in 34 of 44 (77%) patients. Jiang et al. [70] reported that acupuncture in the wrist, ankle, and body could significantly improve the neuropathy symptoms. Zhang et al. [71] reported that among the 32 participants in the acupuncture group, 16 reported “markedly improved”, and 12 reported “improved”. Meanwhile, among the 33 participants in the control group, there were 7 cases of “markedly improved” and 14 cases of “improved”. Tong et al. [72] reported that acupuncture relieved the severity and extent of subjective symptoms compared to the control group. Garrow et al. [74] reported improvements in the LANSS score, VAS pain intensity, MYMOP score, and SF-36 physical component scores. However, the SF-36 bodily pain score did not significantly improve compared to the control. Jeon et al. [75] reported that the TSS and MNSI scores improved. Bailey et al. [76] reported improvement in NTSS-6 scores that acupuncture significantly relieved aching pain, burning pain, tingling and prickling, numbness, and allodynia, but not lancinating pain. Shin et al. [78] reported that the PI-NRS scores significantly improved compared to the control group. The short-form McGill pain questionnaire, sleep interference scores, and the EuroQol-5 dimensions were significantly better in the EA group than in the control group. The percentage of patients who had improved in the patient global impression of change was greater in the EA group than in the control group. Chao et al. [79] reported that acupuncture significantly alleviated the average, worst, and least pain intensities, measured using the NRS. There was also a significant improvement in the quality of life and physical functioning from baseline to week 12 in the acupuncture group. However, the differences between the acupuncture and control groups in the quality of life, physical functioning, and neuropathic symptoms from baseline were not significant. Meyer-Hamme et al. [84] reported improvements in the patient-related outcomes (neuropathic pain, hyperesthesia, cold sensation, heat sensation, burning pain, tingling, muscle cramps, numbness, unsteadiness of gait, impact on daily activities, impact on sleep quality, and frequency of symptoms) in the manual and laser acupuncture groups.

#### 2.3.2. Neuropathy Disability Score (NDS)

The NDS is used to evaluate temperature sensation, pinprick sensation, vibration threshold, and the Achilles reflex. It has a range of 0–8, and each test is rated “0” for a normal response and “1” for an abnormal response. NDS reflects neuropathy disability with good sensitivity [68]. In this review, NDS was assessed in two studies [67,76]; however, no significant differences were observed in both results. Abuaisha et al. [67] reported no significant changes in the NDS after six sessions of manual acupuncture treatment at LI3, SP6, SP9, and ST36 (7.3 ± 0.5 to 7.2 ± 0.7). Similarly, in the study by Bailey et al. [76] manual acupuncture failed to attenuate the NDS (5.2 ± 3.6 to 4.9 ± 3.4).

#### 2.3.3. Vibration Perception Threshold (VPT)

The VPT is considered a valuable assessment tool for neural dysfunction [69]. Two studies [67,72] assessed VPT in patients with diabetic neuropathy. However, the results differed. Abuaisha et al. [67] reported no significant changes in the VPT measured at the great toe after treatment (30.4 ± 1.9 vs. 31.1 ± 2.2 volts). However, Tong et al. [72] reported that the VPT significantly improved from 8.05 ± 3.22 s to 8.56 ± 3.43 s compared to baseline. In their study, the VPT was measured on the medial malleolus in the lower extremities using a handheld biothesiometer. The vibration voltage was increased until the patient could perceive the vibration

#### 2.3.4. Nerve Conduction Velocity

In total, three studies [70,72,84] observed the nerve conduction velocity. Jiang et al. [70] reported that in the wrist–ankle and body acupuncture groups, the CVs of the median nerve and the common peroneal nerve significantly improved after acupuncture treatment compared to baseline. However, in the control group, the changes were not significant. Tong et al. [72] reported that the F-wave minimum latency and MNCV in the tibial nerve significantly improved after acupuncture treatment compared to the baseline group. The FCV in the median nerve also increased. The SNCV of the forearm significantly improved after acupuncture treatment compared to the baseline. Meyer-Hamme et al. [84] reported that the sural SNAP and SNCV were significantly elevated after manual and laser acupuncture. However, the tibial MNCV improved significantly only in the manual acupuncture group. The tibial MNAP did not differ between the groups.

#### 2.3.5. Side Effects

Side effects were reported in five studies [67,74,78,79,84]. In the study by Abuaisha et al. [67], one patient withdrew after two sessions of acupuncture treatment due to discomfort. In Garrow et al.’s study [74], one patient had chest pain related to a chronic heart condition, and the other had exacerbated leg pain after acupuncture treatment. Shin et al. [78] reported 12 adverse effects in both the acupuncture and control groups, but there was no significant difference between the two groups. Chao et al. [79] reported 15 cases of side effects, such as 8 pain, 2 swelling, 2 numbness, 1 nausea, 1 cramp, and 1 palpitation in the left arm. However, none were reported to be serious. Meyer-Hamme et al. [84] reported minor hematomas in approximately 13 out of 60 (22%) participants. A total of 7 out of 180 patients (4%) reported a temporary increase in neuropathic pain and a decrease in hypoesthesia.

#### 2.3.6. Most Used Acupoints

Acupoints Ex-LE10, GB34, SP6, SP9, and ST36 have been used in several clinical studies (Table 3). The most widely used acupoint was SP6 as it has been chosen in seven studies. The second most mentioned acupoint was ST36, which was used in six studies. Ex-LE10, GB34, and SP9 were used in four studies.

## 3. Discussion

Diabetic neuropathy is a serious threat to diabetic patients, and although various treatment methods are available to attenuate this, an optimal drug or treatment method has yet to be identified. In this review, 15 papers that focused on the effect of acupuncture on diabetic neuropathy were included, with ten and five studies being conducted in humans and rodents, respectively. To our knowledge, this was the first study to summarize and analyze the effect of acupuncture on diabetic neuropathy based on data obtained from both humans and rodents. There have been no previous papers on the same subject. Similar papers had slightly different themes. One prior study studied the relationship between acupuncture and neuropathy, but it did not specifically focus on diabetic neuropathy [86]. Another study focused on diabetic neuropathy, but only acupuncture at ST36 has been analyzed [87]. Another study just focused on manual acupuncture and diabetic neuropathy [88]. The other examined evidence of manual acupuncture in the management of diabetic neuropathy [89]. However, in this review, we tried to clarify the effect of acupuncture, including manual acupuncture, EA, and laser acupuncture, and we also tried to investigate the underlying mechanisms of action based on animal studies.

Among the five in vivo studies, four [35,37,45,51] used EA and one [60] used manual acupuncture. Among the clinical trials, eight [67,70,71,72,74,75,76,79] applied manual acupuncture, one used EA [78], and one used both manual and laser acupuncture [84]. The most commonly used acupoint was ST36, which was used in four in vivo studies [35,37,45,51] and six clinical trials [67,71,72,74,75,78]. In the studies conducted on rodents, BL13 and BL20 were the most frequently used acupoints after ST36 [45,60]. In the clinical trials, the acupoints selected more than four times were SP6 (seven times), ST36 (six), and SP9 (four). In a previously published study that analyzed 100 patients with diabetes, the most commonly used acupoints were ST36, SP6, BL20, BL23, and BL13 [90]. It should be noted that ST36 has been proved to be effective against the treatment of various neuropathic pain [91]. In addition, one systematic review suggested that acupuncture on ST36 seems to be more effective in reducing pain and improving nerve conduction velocity [92]. In traditional Chinese medicine, it is believed that ST36 dredges the meridians, removes blood stasis, and promotes blood circulation [90]. ST36, BL23, and SP6 have also been widely used against diabetes mellitus [93]. EA applied to SP9 showed significant thermal and mechanical analgesic effects [94]. Based on these results, we suggest that ST36, BL13, BL20, BL23, SP6, and SP9 acupoints could be used to treat diabetes mellitus and diabetic neuropathy.

In this review, among the five studies that used EA, three [37,45,51] used only lower frequencies (2–3 Hz), whereas the other two [35,78] stimulated the needles with both lower and higher frequencies (2 Hz/100 Hz and 2 Hz/120 Hz, respectively). It is known that low frequencies, such as 2 Hz, can accelerate the release of enkephalin, β-endorphin, and endomorphin. On the other hand, a frequency of 100 Hz could increase the release of dynorphin [53]. In a study by Han, EA stimulation using alternating frequencies of 2 Hz and 100 Hz has been suggested to increase the analgesic effect as it could maximize the opioid release in the brain [93]. Furthermore, in all studies, acupuncture was applied for more than 20 min. This may be due to the report that acupuncture applied 20–30 min resulted in longer-lasting outcomes [95].

In one study [84], both manual and laser acupuncture were applied to patients. Although both acupunctures succeeded in improving the sural SNAP and SNCV compared to baseline, only manual acupuncture significantly improved tibial MNCV in patients with diabetic neuropathy. Based on these results, authors have stated that despite the improvements in patient-related outcomes, the effect of laser acupuncture remains below those of manual acupuncture. However, in several studies, laser acupuncture has been considered as an alternative form of traditional acupuncture as it was reported to be effective against pain. Increasing evidence has suggested that the use of laser acupuncture can relieve myofascial pain, chronic tension headache, edema, and hyperalgesia [86,87]. Moreover, spontaneous pain and thermal hyperalgesia induced by neuropathic pain was also shown to be alleviated [87]. The activation of the endogenous opioidergic and serotonergic systems have been suggested as the underlying mechanism of action of laser acupuncture [88].

Moreover, the acupuncture treatment period was assessed, and although it is difficult to make conclusions because the treatment modalities were all different, the results suggest that a single acupuncture treatment may not be sufficient in attenuating diabetic neuropathy. Drugs conventionally used against diabetic neuropathy, such as gabapentin and pregabalin, are also recommended for more than 8 weeks [96]. Indeed, in the study by Zhou et al. [37], in which acupuncture was administered briefly for 1 week, myelin disruption and dissolute axoplasm of nerve fibers were still observed, although the mechanical pain was reduced. However, in the study by Pan et al. [45], EA treatment, which was applied for 72 sessions over 12 weeks, ameliorated the myelin sheath and nerve fiber damage. Although the acupoints used were different (ST36, BL60 vs. BL13, BL20, BL23, LI4, LR3, ST36, SP6), the relatively longer treatment period applied in the study of Pan et al. (12 weeks vs. 1 week) may have affected the results. Moreover, in all clinical trials, acupuncture was administered for more than 1 month, except for the study by Tong et al. [72], where 15 sessions of acupuncture significantly relieved the subjective pain symptoms. However, further research is needed to clarify whether the treatment period or the number of sessions is critical in attenuating diabetic neuropathy.

**Figure 1:**
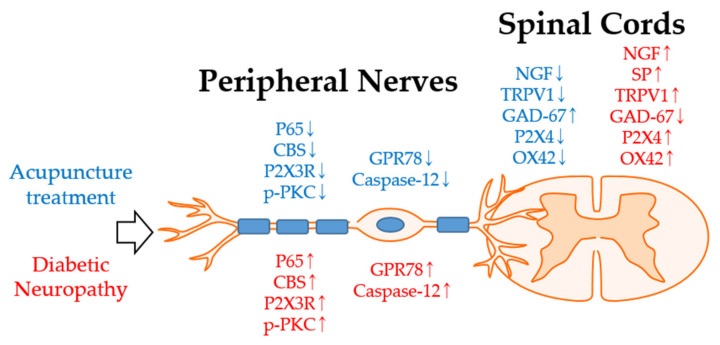
Analgesic mechanism of acupuncture against diabetic neuropathy in the peripheral nerves and spinal cord in rats. Intraperitoneal injection of STZ induces diabetic neuropathy (**red**), and acupuncture treatments in various acupoints attenuate neuropathy (**blue**) in rats. P65, CBS, P2X3R p-PKC GPR78, and caspase-12 are upregulated after STZ injection. However, acupuncture treatment in various acupoints upregulated the downregulated molecules. Furthermore, in the spinal cord, there is an STZ injection-induced upregulation of the NGF, SP, TRPV1, P2X4, and OX42 upregulation and downregulation of the GAD. Abbreviations: CBS, cystathionine β synthase; GAD-67, glutamic acid decarboxylase-67; GPR78, G-protein coupled receptor 78; NGF, nerve growth factor; OX42, microglia marker; P2X3, P2X purinoceptor 3; P2X4, P2X purinoceptor 4; p-PKC, p-protein kinase C; SP, substance P; TRPV1, transient receptor potential vanilloid 1.

In all five animal studies, STZ was injected to mimic diabetic neuropathy in rodents. STZ is widely used to produce a model of type 1 and 2 diabetes by damaging the pancreatic islet β-cells [97]. Various pathways have been implicated in the mechanism of action of acupuncture. Three [35,37,45] and two studies [51,60] observed the effect of acupuncture on the DRG and spinal cord, respectively. In the DRG, the effect of acupuncture analgesia may be related to NF-κB signaling, PKC-dependent P2X3 signaling, and ERS apoptosis. Shi et al. [35] observed that CBS was decreased by NF-κB inhibition. Furthermore, EA treatment suppressed both p65 and CBS expressions in the DRG. Zhou et al. [37] observed that EA suppressed the upregulation of p-PKC expression in the DRG and was related to the analgesic effect of EA. Pan et al. [45] showed that the upregulation of GPR78 and caspase-12 in diabetic neuropathy is blocked by EA. In the spinal cord, the analgesic effect of acupuncture may be related to NGF signaling, P2X4 expression, and inflammation of the spinal cord. Manni et al. [51] highlighted that increased NGF and TrkA levels after STZ treatment in the spinal cord were lowered after acupuncture treatment. Increased SP in the skin and TRPV1 in the spinal cord after STZ treatment was eventually lowered, while the decreased glutamic acid decarboxylase-67 levels in the spinal cord after STZ treatment were restored after acupuncture treatment. Tang et al. [60] observed that increased P2X4 and microglia marker expressions by STZ treatment were counteracted by acupuncture (Figure 1).

One animal study [45] and four clinical trials [70,72,78,84] observed a change in nerve conduction velocity after acupuncture treatment in the diabetic state. Pan et al. [45] demonstrated that EA could increase the downregulated MCV and sensory conduction velocity of the sciatic nerve after STZ injection. Jiang et al. [70] showed that the MCV of the median nerve and common peroneal nerve significantly improved after acupuncture treatment. Tong et al. [72] also demonstrated significant improvements in the MNCV and FCV in the tibial and median nerves, respectively. They also reported that the SNCV of the forearm significantly improved after acupuncture treatment. Furthermore, Meyer-Hamme et al. [84] demonstrated that both manual and laser acupuncture could increase the SNAP and SNCV of the sural nerve and the MNCV of the tibial nerve. However, in their study, the MNAP of the tibial nerve was not different from that of the control. Shin et al. [78] reported no significant differences in nerve conduction.

Five studies [67,74,78,79,84] reported side effects, including minor hematomas, temporary pain, swelling, numbness, nausea, cramps, and palpitations. However, none were serious. Taken together, this suggests that acupuncture could provide safe and effective treatment options with relatively mild adverse effects [98] compared to conventional drug medicine with high rates of adverse events [99].

In conclusion, based on the results obtained from all the included studies, we suggest that acupuncture could be considered a useful treatment method for diabetic neuropathy. However, more well-designed experimental and clinical trials should be conducted to better assess its effects on diabetic neuropathy. We believe that this review will help researchers in the field of acupuncture and diabetic neuropathy to better understand its mechanism in the future.

## 4. Methods

All studies on acupuncture (manual, electro-, and laser acupuncture) and diabetic neuropathy in PUBMED from the National Library of Medicine and Google Scholar were collected. Extensive searches were undertaken for articles written in English since non-English studies were excluded. Studies electronically published until the end of April 2021 were included. The literature search was performed using the following keywords: “acupuncture”, “electro-acupuncture”, “laser acupuncture”, and “diabetic neuropathy.” The total number of articles after the initial search was 199, including 29 clinical trials. Subsequently, the duplicates, bibliographies, study protocols, and non-English studies were excluded. In total, ten clinical trials and five in vivo studies were included in the study.

## Figures and Tables

**Table 2:** Summary of Studies Conducted in Humans.

Author	Characteristics of Patients	Acupuncture Treatment ① Acupuncture Types ② Acupoints ③ Retention Time ④ Treatment Duration (Sessions) ⑤ Conventional Treatment ⑥ Control to Acupuncture		Outcomes	
				Patients-Related Outcomes	Nerve-Function Outcomes
Abuaisha et al., 1998 [67]	Participants: *n =* 44	①	Manual acupuncture	Improved in primary (74.9 ± 2.4 to 44.5 ± 4.3) and secondary (70.3 ± 2.9 to 43.5 ± 4.2) symptoms: 34/44 (77%) Complete symptoms relief after treatment: 7/34 (21%) No significant change in NDS (7.3 ± 0.5 to 7.2 ± 0.7)	No significant change in VPT (30.4 ± 1.9 to 31.1 ± 2.2 volts)
		②	LI3, SP6, SP9, ST36		
		③	20 min (first week: 5 min)		
		④	10 weeks (6)		
		⑤	Analgesics, tricyclic drugs, anticonvulsants (63%)		
		⑥	-		
Jiang et al., 2006 [70]	Participants: *n =* 90 Wrist-ankle acupuncture: *n =* 30 Body acupuncture: *n =* 30 Control: *n =* 30	①	Manual acupuncture	Wrist-ankle acupuncture: 56.67% markedly relieved and 36.67% improved Body acupuncture: 56.67% markedly relieved and 33.33% improved Control: 23.33% markedly relieved and 40.00% improved	Wrist-ankle acupuncture: Improved CV of median (42.12 ± 3.80 → 46.87 ± 5.57 m/s) & common peroneal nerve (41.42 ± 4.47 → 45.45 ± 4.82 m/s) Body acupuncture: Improved CV of median (42.17 ± 4.51 → 45.48 ± 4.92 m/s) & common peroneal nerve (42.12 ± 4.63 → 45.37 ± 4.90 m/s) Control: No change in CV of median (42.22 ± 4.90 → 43.15 ± 5.24 m/s) & common peroneal nerve (42.04 ± 4.53 → 43.91 ± 5.51 m/s)
		②	Wrist-ankle acupuncture: upper 2 and lower 2 Body acupuncture: SP6, SP10, KI3, LI11, GB34 Additional acupoints regarding to symptoms applied to both groups		
		③	15–30 min		
		④	25 days (21)		
		⑤	-		
		⑥	VB_1_ and VB_12_, i.m.		
Zhang et al., 2010 [71]	Participants: *n =* 65 Acupuncture: *n =* 32 Control: *n =* 33	①	Manual acupuncture	Acupuncture: 16 markedly relieved, 12 improved, 4 failed Control: 7 markedly relieved, 14 improved, 12 failed	-
		②	BL18, BL20, BL23, BL58, ST36, SP6, SP3, CV6, CV4, ST40, GB34, Ex-B3 + acupoints regarding to symptoms		
		③	25 min		
		④	3 months (70)		
		⑤	-		
		⑥	Inositol, p.o., 2 g/day		
Tong et al., 2010 [72]	Participants: *n =* 63 Acupuncture: *n =* 42 Control: *n =* 21	①	Manual acupuncture	Improved numbness of lower extremities Improved spontaneous pain of lower extremities Improved rigidity in upper extremities Improved alterations in temperature perception in lower extremities	Improved F-wave minimum latency in tibial nerve (52.6 ± 0.5 → 53.0 ± 0.3 m/s) Improved MNCV in tibial nerve (39.5 ± 0.5 → 40.2 ± 3.9 m/s) Improved FCV in median nerve (55.6 ± 0.4 → 56.5 ± 0.5 m/s) Improved SNCV of forearm (47.8 ± 0.5 → 48.3 ± 0.7 m/s) Improved VPT (8.05 ± 3.22 → 8.56 ± 3.43 s)
		②	LI4, ST40, LI11, ST36, SP6		
		③	30 min		
		④	15 days (15)		
		⑤	-		
		⑥	0.3 cm (vs. 1.2–2.3 cm) needles insertion without stimulation		
Garrow et al., 2014 [74]	Participants: *n =* 45 Acupuncture: *n =* 24 Control: *n =* 21	①	Manual acupuncture	Improved LANSS score (14.3 ± 6.4 → 13.6 ± 7.2) Improved VAS pain intensity (73 ± 24 → 58 ± 26) Improved MYMOP score (4.3 ± 1.2 → 3.4 ± 1.3) Improved SF-36 physical component score (40.7 ± 13.2 → 39.2 ± 14.0) No change in SF-36 bodily pain score (37.7 ± 27.4 → 40.2 ± 20.2)	-
		②	LR3, KI3, SP6, ST36		
		③	30 min		
		④	10 weeks (10)		
		⑤	-		
		⑥	Sham acupuncture (blunt and slides into the handle)		
Jeon et al., 2015 [75]	Participants: *n =* 9	①	Manual acupuncture	No significant change in TSS score (7.99 ± 3.55 → 4.95 ± 4.41) No significant change in MNSI score (6.33 ± 1.31 → 4.33 ± 3.00)	-
		②	Ex-LE10, LR3, GB34, GB39, GB41, ST36, SP6, SP9		
		③	21–28 min		
		④	4 weeks (12)		
		⑤	-		
		⑥	-		
Bailey et al., 2017 [76]	Participants: *n =* 25	①	Manual acupuncture	Improved NTSS-6 scores: aching pain (2.4 ± 0.6 → 1.6 ± 0.5) burning pain (1.7 ± 0.7 → 1.0 ± 0.6) tingling and prickling (2.2 ± 0.5 → 1.2 ± 0.6) numbness (1.7 ± 0.6 → 1.0 ± 0.6) allodynia (1.9 ± 0.6 → 1.2 ± 0.7) No significantly different NTSS-6 scores: lancinating pain (2.0 ± 0.6 → 1.6 ± 0.6) NDS (5.2 ± 3.6 → 4.9 ± 3.4)	-
		②	EX-LE10, ST32, ST37, ST42, SP7, SP9, KI1, KI3, KI9, LR4, LR7, GB34, GB37		
		③	30 min		
		④	10 weeks (10)		
		⑤	-		
		⑥	-		
Shin et al., 2018 [78]	Participants: *n =* 126 Acupuncture: *n =* 63 Control: *n =* 63	①	EA (2/120 Hz)	Improved PI-NRS scores (−0.67 [95% CI−1.29 to −0.06] vs. control) at week 9 Improved short-form McGill pain questionnaire, sleep interference scores, and the EuroQol-5 dimensions at week 9	No significant difference in nerve conduction velocity
		②	ST36, GB39, SP9, SP6, LR3, GB41 + additional acupoints regarding to symptoms (Ex-LE10)		
		③	-		
		④	8 weeks (16)		
		⑤	Anti-diabetes and rescue medication allowed (acetaminophen 500 mg, max 3000 mg/day)		
		⑥	No EA treatment		
Chao et al., 2019 [79]	Participants: *n =* 40 Acupuncture (1/week): *n =* 14 Acupuncture (2/week): *n =* 12 Control: *n =* 14	①	Manual acupuncture	Improved NRS score (between-group differences): Average pain intensity (−1.86 (week 6), −2.06 (week 12)) Worst pain intensity (−1.88 (week 6), −2.34 (week 12)) Least pain intensity (−1.24 (week 6), −1.46 (week 12))	-
		②	Jing well and shu stream acupoints + acupoints regarding to symptoms (8–12 acupoints)		
		③	20–40 min.		
		④	12 weeks (12 and 24)		
		⑤	Antidepressants, opiates, and anticonvulsants		
		⑥	No acupuncture treatment		
Meyer-Hamme et al., 2020 [84]	Participants *n =* 120 Acupuncture *n =* 60 Laser acupuncture *n =* 60 Control *n =* 60	①	Manual and laser acupuncture	Improved 12/12 items of patient-related outcomes in acupuncture group Improved 11/12 items of patient-related outcomes (exclusion: hyperesthesia) in laser acupuncture group Improved 9/12 of patient-related outcomes (exclusion: hyperesthesia, heat sensation, muscle cramps) in control	Improved sural SNAP (μV) in all three groups Improved sural SNCV (m/s) in the manual and laser acupuncture group Improved tibial MNCV (m/s) in the manual acupuncture group No significant difference in tibial MNAP (mV) in all group
		②	Ex-LE10, Ex-LE12, ST34		
		③	20 min		
		④	10 weeks (10)		
		⑤	-		
		⑥	Laser acupuncture without laser		

Abbreviations: Ach: acetylcholine; CV: conduction velocity; FCV: F-wave conduction velocity; HbA1c: glycosylated hemoglobin; LANSS: Leeds assessment of neuropathic symptoms and signs; LDF: laser Doppler fluxmetry; LDS: neuropathy disability score; MNAP: motor nerve action potential; MNCV: motor nerve conduction velocity; MNSI: Michigan neuropathy screening instrument; MYMOP: Measure Yourself Medical Outcome Profile; NDS: neuropathy disability score; NRS: numerical rating scale; NS: non-significant; NTSS: neuropathy total symptom scale; PI-NRS: pain intensity numerical rating scale; SF-36: Short form 36; SNAP: sensory nerve action potential; SNCV: sensory nerve conduction velocity; SNP: Sodium nitroprusside; SPS: sleep problem scale; TSS: total symptom score; VAS: Visual analog scale; VB: Vitamin B; VPT: vibration perception threshold.

**Table 3:** Anatomical Location of Acupoints.

Acupoints	Anatomical Location	Number of Studies Used	References
Ex-LE10 (Bafeng)	Between each proximal phalanx	4	[75,76,78,84]
GB34 (Yanglingquan)	On the fibular aspect of the leg, in the depression anterior, and distal to the head of the fibula.	4	[70,71,75,76]
SP6 (Sanyinjiao)	On the tibial aspect of the leg, posterior to the medial border of the tibia, 3 B-cun [85] superior to the prominence of the medial malleolus.	7	[67,70,71,72,74,75,78]
SP9 (Yinlingquan)	On the tibial aspect of the leg, in the depression between the inferior border of the medial condyle of the tibia, and the medial border of the tibia.	4	[67,75,76,78]
ST36 (Zusanli)	On the anterior aspect of the leg, on the line connecting ST35 with ST41, 3 B-cun inferior to ST35	6	[67,71,72,74,75,78]
